# The Development, Implementation, and Evolution of an Emergency Medicine Ultrasound-guided Regional Anesthesia Curriculum

**DOI:** 10.5811/westjem.59793

**Published:** 2023-12-06

**Authors:** Sally Graglia, Derek Harmon, Barbie Klein

**Affiliations:** * University of California San Francisco, Department of Emergency Medicine, San Francisco, California; † Zuckerberg San Francisco General Hospital, San Francisco, California; ‡ University of California San Francisco, School of Medicine, Department of Anatomy, San Francisco, California

## Abstract

**Introduction:**

Despite the inclusion of both diagnostic and procedural ultrasound and regional nerve blocks in the original Model of the Clinical Practice of Emergency Medicine (EM), there is no recommended standardized approach to the incorporation of ultrasound-guided regional anesthesia (UGRA) education in EM training.

**Methods:**

We developed and implemented a structured curriculum for both EM residents and faculty to learn UGRA in a four-hour workshop. Each Regional Anesthesia Anatomy and Ultrasound Workshop was four hours in length and followed the same format. Focusing on common UGRA blocks, each workshop began with an anatomist-led cadaveric review of the relevant neuromusculoskeletal anatomy followed by a hands-on ultrasound scanning practice for the blocks led by an ultrasound fellowship-trained EM faculty member, fellow, or a postgraduate year (PGY)-4 resident who had previously participated in the workshop. Learners identified the relevant anatomy on point-of-care ultrasound and reviewed how to conduct the blocks. Learners were invited to complete an evaluation of the workshop with Likert-scale and open-ended questions.

**Results:**

In the 2020 academic year, six regional anesthesia anatomy and ultrasound workshops occurred for EM faculty (two sessions, N = 24) and EM residents (four sessions, N = 40, including a total of five PGY4s, 10 PGY3s, 12 PGY2s, and 13 PGY1s). Workshops were universally well-received by both faculty and residents. Survey results found that 100.0% of all responding participants indicated that they were “very satisfied” with the session. All were likely to recommend this session to a colleague and 95.08% of participants believed the session should become a required component of the EM curriculum.

**Conclusion:**

The use of UGRA is increasing, and and it critical in EM. An interdisciplinary approach in collaboration with anatomists on an interactive, nerve block workshop incorporating both gross anatomy review and hands-on scanning was shown to be well-received and desired by both EM faculty and residents.

## BACKGROUND

There is currently no standardized educational approach to teaching ultrasound-guided regional anesthesia (UGRA) in emergency medicine (EM) training.^1^ The use of point-of-care ultrasound (POCUS) is pervasive in EM, but novel POCUS applications are constantly challenging previously accepted “standards of care.” The original 2001 Model of the Clinical Practice of Emergency Medicine included both diagnostic and procedural ultrasound and regional anesthesia.^2^ While POCUS use and regional anesthesia were recognized as critical components of EM training, the opioid epidemic and emergency department (ED) presentations surged.^3^ Shown to decrease the use of opioids in pain management, UGRA has a potential role in combating the opioid pandemic.^4^ ^–^ ^8^

## OBJECTIVES

Our goal was to create a standardized EM UGRA curriculum by providing a conceptual foundation, hands-on practice, and educational resource for common nerve blocks. The study was reviewed by the institutional review board and classified as exempt (Protocol #19-29811).

## CURRICULAR DESIGN

We developed this curriculum following Kern’s six-step approach:^9^

1. Problem identification
  First, the need for UGRA training in EM was identified. Literature presents the utility of specific blocks or generalized need for UGRA education but not a comprehensive framework.
2. Targeted needs assessment
  Exposure to UGRA by EM residents is dependent on the specific patients who present to the ED, whether the residents are on ultrasound rotation, and the comfort level of their supervising physician. We identified the need for a comprehensive UGRA curriculum, including the following nerve blocks:
  - Superficial cervical plexus
  - Interscalene
  - Supraclavicular
  - Radial
  - Median
  - Ulnar
  - Serratus anterior
  - Fascia iliaca (traditional and bowtie)
  - Femoral
  - Saphenous
  - Sciatic
  - Tibial nerve (ankle)
3. *Goals/Objectives*
  For each nerve block, the learning objectives included the following:
  - Common indications
  - Anticipated area of anesthesia
  - Anesthetic used
  - Set-up
  - Obtaining an accurate sonographic image to perform the block
  - Critical anatomy
  - Additional relevant anatomy
  - Technique
  - Associated risks
4. *Educational strategies*
  This session used several evidence-based learning strategies. Coupling the visualization of anatomic structures through interactive cadaveric review with the corresponding sonographic identification, the three-dimensional anatomy was translated to the two-dimensional sonographic image. Repetition and review were embedded throughout the session as every learner had the opportunity to discuss and practice each specific block and observe their colleagues multiple times. Real-time discourse between anatomists (faculty within the Department of Anatomy who hold a doctoral degree in anatomical sciences) and emergency physicians ensured active learning. Finally, and perhaps most importantly, this session provided a comprehensive mental framework, equipping learners with the knowledge and preparation necessary to perform each block.

### Objective Structured Clinical Examination (OSCE) Development

**Figure 1.**
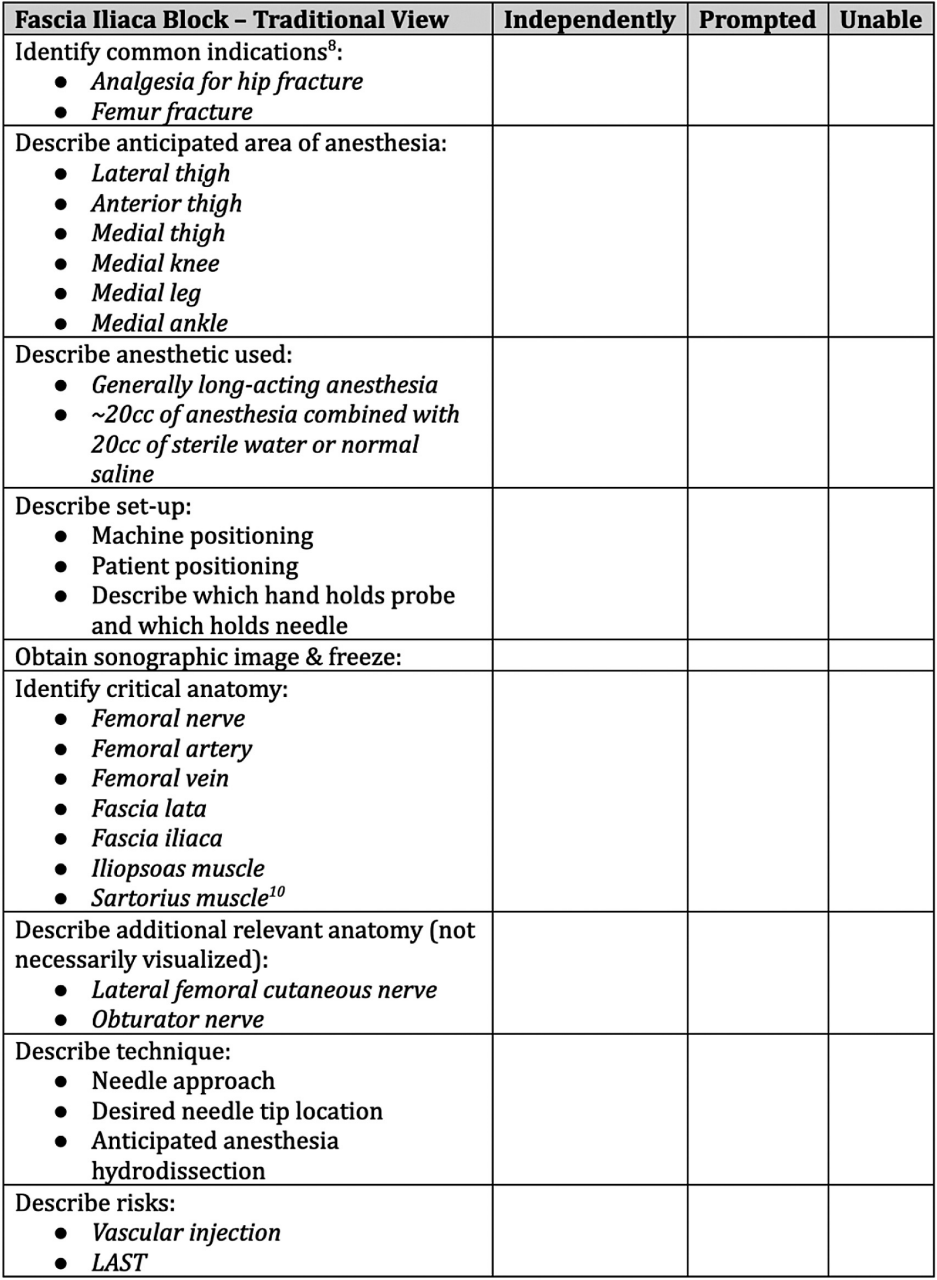
Example of the OSCE for a single block, the fascia iliaca block (traditional view), outlining common indications, anticipated area of anesthesia, anesthetic used, set-up, sonographic image, critical anatomy, relevant anatomy, technique, and risks.

With the intention to assess competency of session attendees in the future, an OSCE was developed and piloted. While OSCEs exist for clinical skills in EM^10^ and for ultrasound-guided procedures,^11^ ^,^ ^12^ an OSCE specific for ultrasound-guided regional anesthesia had not been reported at the time of our research. The OSCE was developed by two of the authors combining their respective expertise in gross anatomy (DH) and clinical application of nerve blocks (SG). The OSCE was revised through an iterative consensus process after being reviewed by three ultrasound fellowship-trained EM faculty who are experts in regional anesthesia. The OSCE was used as a real-time reference sheet for learners during these sessions and provided as an educational resource for participants in the future. An example of the OSCE for a single block is presented in Figure 1 and is included entirely as Supplemental File A.

5. *Implementation*
  The session was piloted during the 2019-2020 academic year with a cohort of EM residents rotating on the pain elective and implemented as a mandatory component of the EM curriculum in the 2020-2021 academic year. As a part of the mandatory EM curriculum, the session was offered separately to each residency class and twice to EM faculty.
6. *Evaluation of effectiveness*
  Both residents and faculty were asked to evaluate the session. The evaluation included questions assessing participants’ overall satisfaction with the session, likelihood of recommending the session to colleagues, interest in attending a similar session in the future, perceived length of the session, and whether the workshop should be a required component of the EM curriculum. Additionally, four open-ended questions asked what, if anything, participants learned during the workshop that could be applied to their clinical practice, what was most beneficial, how the workshop could be improved, and any other topics of interest to learn within the anatomy laboratory.

### Implementation Phase

In the 2020 academic year, six regional anesthesia anatomy and ultrasound workshops occurred for EM residents (four sessions, N = 40, including a total of 5 PGY4s, 10 PGY3s, 12 PGY2s, and 13 PGY1s) and EM faculty (two sessions, N = 24). Each session was approximately four hours in length, followed the same format, and was led by an ultrasound-trained emergency physician and an anatomist.

The entire workshop was hosted in the anatomy laboratory and focused on the nerve blocks described in the *targeted needs assessment.* Workshops began with an anatomist-led review of the relevant upper extremity neuromusculoskeletal anatomy on a pre-dissected cadaveric donor followed by a hands-on ultrasound scanning practice session of the same blocks led by an ultrasound-trained emergency physician. For each specific nerve block, learners identified the relevant anatomy on POCUS and reviewed how to conduct an optimal scan and block. Learners repeated this process for each block until each learner felt comfortable.

Following the upper extremity section, the workshop repeated the format for the lower extremity nerve blocks listed in the *targeted needs assessment.* Following the anatomy review and ultrasound training for the lower extremity, learners were asked to complete an evaluation.

## IMPACT/EFFECTIVENESS

Of 40 residents and 24 faculty who participated, 39 residents (97.5%) and 22 faculty (91.7%) completed an evaluation. The workshop sessions were universally well-received by both EM residents and faculty (Table 1). Open response comments noted several benefits of the session and areas for improvement.

**Table 1.** Evaluation data for regional anesthesia anatomy and ultrasound workshops.

Question	Answers	Faculty (N = 22)	Residents (N = 39)	Combined (N = 61)
How would you rate your overall satisfaction with the session?	Very satisfied	100.0% (N = 22)	100.0% (N = 39)	100.0% (N = 61)
How likely are you to recommend this session to your colleagues?	Very likely	100.0% (N = 22)	100.0% (N = 39)	100.0% (N = 61)
Would you be interested in attending a session like this again?	Yes	95.45% (N = 21)	100.0% (N = 39)	98.36% (N = 60)
	No	4.55% (N = 1)	-----	1.64% (N = 1)
Do you believe this session should be a required component of the EM curriculum?	Yes	95.45% (N = 21)	94.87% (N = 37)	95.08% (N = 58)
	No	-----	5.13% (N = 2)	3.28% (N = 2)
	Offered, but not required^*^	4.55% (N = 1)	-----	1.64% (N = 1)
How would you describe the length of this session?	Perfect	95.45% (N = 21)	82.05% (N = 32)	86.89% (N = 53)
	Too long	4.55% (N = 1)	12.82% (N = 5)	9.84% (N = 6)
	Between perfect/too long^*^	-----	5.13% (N = 2)	3.28% (N = 2)

* Some participants wrote in additional answers for questions that were between Likert scale units.

*EM*, emergency medicine.

## LIMITATIONS

This study is not without limitations. This session focused on building foundational knowledge rather than motor skills; therefore, participants were unable to practice needle insertion necessary to perform ultrasound-guided procedures. Due to time constraints, assessment of learner competence in independently performing the procedures using a pre- and post-OSCE was not conducted. Additionally, because of the way residents log procedures, the number of times they had performed these procedures prior to and following this session were unavailable for analysis. Similarly, the effect of this session on clinical outcomes in patient care—the gold standard for any educational intervention targeting a specific clinical skill— was beyond the scope of this initial implementation. Finally, this session is dependent upon collaboration with anatomy educators with access to an anatomy laboratory and dissected cadavers demonstrating the relevant gross anatomy which may not be feasible for all EM residencies.

### Future Directions

With regard to future iterations, most immediately we will make changes to the session based on collective feedback. The most consistent feedback from learners was the desire to practice needle visualization and anesthetic injection for these blocks on cadavers or phantoms. Going forward, sessions will employ hands-on manipulation of the ultrasound transducer and needle placement and insertion on either unembalmed cadavers or on phantom simulators to enable learners to build the skill of hand-eye coordination necessary to perform these procedures. Future sessions will incorporate additional innovative blocks as they emerge, such as the erector spinae plane block.^13^ On a competency level, next steps will focus on evaluating the reliability and validity evidence for the OSCE as an assessment tool. On a program level, steps can be taken to incorporate the OSCE to assess learners’ ability to independently perform these UGRA procedures. As this session has been incorporated into the structure of the EM residency curriculum, learners may be assessed longitudinally throughout their residency training, using this OSCE to demonstrate independence in performing these procedures.

## CONCLUSION

Given the lack of an existing standardized UGRA curriculum in EM training, we chose to use Kern’s six-steps of curriculum development to construct a curriculum that can be replicated at other institutions. A novel element to the success of our session was bringing clinicians back to the anatomy laboratory to visualize the gross anatomy and the corresponding sonographic identification for a comprehensive review of UGRA.

We attribute the success of this session to several factors. First, feedback was elicited after each session and considered to enhance session effectiveness. Implemented first as a pilot session in the 2019–2020 academic year, the OSCE was edited and the structure of the session was adapted for the 2020–2021 academic year. Second, by using Kern’s model, we provided a level of structure to a previously unstructured skill that allowed learners to adopt a mental model through which they could approach UGRA in their own practice. The structure of the in-person session is directly reflective of the structure of the OSCE, reinforcing the framework. Third, this session was financially feasible. Because our institution’s School of Medicine implements cadaveric-based instruction, this session capitalized on already having access to cadavers; thus, there was no additional cost to the ED to host this session. Additionally, the preservation of cadaveric tissue enabled the continued use of the prosections for subsequent workshops.

## Supplementary Information




