# Supplementary material for: The Development, Implementation, and Evolution of an Emergency Medicine Ultrasound-guided Regional Anesthesia Curriculum
Source: West J Emerg Med. 2023 Dec 6;25(1):117–21. doi: 10.5811/westjem.59793 (PMC10777173; doi:10.5811/westjem.59793)
Supplement: Supplementary file 1 [file wjem-25-117-s001.docx]

**Regional Anesthesia: Observed Structured Clinical Exam**

Trainee: ______________________________________ Evaluator: ____________________________________

(Circle one) pre-OSCE or post-OSCE Date: __________________________________________

| **Superficial Cervical Plexus** | **Independently** | **Prompted** | **Unable** |
| --- | --- | --- | --- |
| Identify common indications^1^:   - *Clavicular fractures* - *Internal jugular vein central catheter placement* - *Neck abscesses* |  |  |  |
| Describe anticipated area of anesthesia:   - *Inferior earlobe* - *Angle of the mandible to the clavicle* - *Clavicle* |  |  |  |
| Describe anesthetic used:   - *Generally short-acting anesthesia* - *~5cc* |  |  |  |
| Describe set-up:   - Machine positioning - Patient positioning - Describe which hand holds probe and which holds needle |  |  |  |
| Obtain sonographic image & freeze: |  |  |  |
| Identify critical anatomy:   - *Sternocleidomastoid muscle* - *Cervical plexus/cervical nerves* |  |  |  |
| Describe additional relevant anatomy (not necessarily visualized): |  |  |  |
| Describe technique:   - Needle approach - Desired needle tip location - Anticipated anesthesia hydrodissection |  |  |  |
| Describe risks: |  |  |  |

| **Interscalene Nerve Block** | **Independently** | **Prompted** | **Unable** |
| --- | --- | --- | --- |
| Identify common indications^2^:   - *Shoulder dislocations* - *Deltoid abscesses* |  |  |  |
| Describe anticipated area of anesthesia:   - *Lateral shoulder* - *Lateral arm* - *Lateral forearm* - *Lateral hand (lateral aspect of thumb)* |  |  |  |
| Describe anesthetic used:   - *Depends on why you’re performing the block* - *~10cc* |  |  |  |
| Describe set-up:   - Machine positioning - Patient positioning - Describe which hand holds probe and which holds needle |  |  |  |
| Obtain sonographic image & freeze: |  |  |  |
| Identify critical anatomy:   - *Internal jugular vein* - *Common carotid artery* - *Sternocleidomastoid muscle* - *Anterior scalene muscle* - *Nerve trunks (Superior trunk C5/C6, Middle trunk C7)* - *Middle scalene muscle* |  |  |  |
| Describe additional relevant anatomy (not necessarily visualized):   - *Subclavian artery* - *Sympathetic trunk* - *Phrenic nerve* |  |  |  |
| Describe technique:   - Needle approach - Desired needle tip location - Anticipated anesthesia hydrodissection |  |  |  |
| Describe risks:   - *Phrenic nerve paralysis (Hemidiaphragmatic paralysis)* - *Horner’s syndrome* |  |  |  |

| **Supraclavicular Nerve Block** | **Independently** | **Prompted** | **Unable** |
| --- | --- | --- | --- |
| Identify common indications^3^:   - *Forearm abscesses* |  |  |  |
| Describe anticipated area of anesthesia:   - *Lateral shoulder* - *Lateral arm* - *Lateral forearm* - *Hand* - *Medial forearm* |  |  |  |
| Describe anesthetic used:   - *Depends on why you’re performing the block* - *~10cc* |  |  |  |
| Describe set-up:   - Machine positioning - Patient positioning - Describe which hand holds probe and which holds needle |  |  |  |
| Obtain sonographic image & freeze: |  |  |  |
| Identify critical anatomy^3^:   - *Middle scalene muscle* - *Subclavian artery* - *Brachial plexus* - *First rib* - *Pleura/pleural interface* |  |  |  |
| Describe additional relevant anatomy (not necessarily visualized): |  |  |  |
| Describe technique:   - Needle approach - Desired needle tip location - Anticipated anesthesia hydrodissection |  |  |  |
| Describe risks:   - *Pneumothorax* - *Subclavian artery injection* |  |  |  |

| **Forearm (Radial nerve)** | **Independently** | **Prompted** | **Unable** |
| --- | --- | --- | --- |
| Identify common indications:   - *Hand lacerations* |  |  |  |
| Describe anticipated area of anesthesia^4^:   - *Dorsal surface of index, long, and radial aspect of ring finger proximal to the distal interphalangeal (DIP) joints* - *Radial aspect of thumb* |  |  |  |
| Describe anesthetic used:   - *Generally short-acting anesthesia* - *~5cc* |  |  |  |
| Describe set-up:   - Machine positioning - Patient positioning - Describe which hand holds probe and which holds needle |  |  |  |
| Obtain sonographic image & freeze: |  |  |  |
| Identify critical anatomy:   - *Radial artery* - *Radial nerve* |  |  |  |
| Describe additional relevant anatomy (not necessarily visualized): |  |  |  |
| Describe technique:   - Needle approach - Desired needle tip location - Anticipated anesthesia hydrodissection |  |  |  |
| Describe risks:   - *Radial artery injection* |  |  |  |

| **Forearm (Median nerve)** | **Independently** | **Prompted** | **Unable** |
| --- | --- | --- | --- |
| Identify common indications:   - *Hand lacerations* |  |  |  |
| Describe anticipated area of anesthesia^4^:   - *Dorsal aspect of digits 2, 3, and radial aspect of 4 distal to the distal interphalangeal (DIP) joints* - *Radial/lateral aspect of volar hand and volar/palmar surface of thumb, index, long, and radial/lateral side of ring finger* |  |  |  |
| Describe anesthetic used:   - *Generally short-acting anesthesia* - *~5cc* |  |  |  |
| Describe set-up:   - Machine positioning - Patient positioning - Describe which hand holds probe and which holds needle |  |  |  |
| Obtain sonographic image & freeze: |  |  |  |
| Identify critical anatomy:   - *Median nerve* |  |  |  |
| Describe additional relevant anatomy (not necessarily visualized):   - *Flexor digitorum superficialis* - *Flexor digitorum profundus* |  |  |  |
| Describe technique:   - Needle approach - Desired needle tip location - Anticipated anesthesia hydrodissection |  |  |  |
| Describe risks: | - | - | - |

| **Forearm (Ulnar nerve)** | **Independently** | **Prompted** | **Unable** |
| --- | --- | --- | --- |
| Identify common indications:   - *Hand lacerations* |  |  |  |
| Describe anticipated area of anesthesia^4^:   - *Ulnar/medial aspect of dorsal and volar (palmar) hand including hypothenar eminence, fifth digit, and ulnar/medial aspect of ring finger* |  |  |  |
| Describe anesthetic used:   - *Generally short-acting anesthesia* - *~5cc* |  |  |  |
| Describe set-up:   - Machine positioning - Patient positioning - Describe which hand holds probe and which holds needle |  |  |  |
| Obtain sonographic image & freeze: |  |  |  |
| Identify critical anatomy:   - *Ulnar artery* - *Ulnar nerve* |  |  |  |
| Describe additional relevant anatomy (not necessarily visualized): | - | - | - |
| Describe technique:   - Needle approach - Desired needle tip location - Anticipated anesthesia hydrodissection |  |  |  |
| Describe risks:   - *Ulnar artery injection* |  |  |  |

| **Serratus Plane Block** | **Independently** | **Prompted** | **Unable** |
| --- | --- | --- | --- |
| Identify common indications:   - *Anterolateral rib fractures* |  |  |  |
| Describe anticipated area of anesthesia^5,6^:   - *Lateral cutaneous branches of the thoracic intercostal nerves (T2-T12)* |  |  |  |
| Describe anesthetic used:   - *Generally long-acting anesthesia* - *~20cc of anesthesia combined with 20cc of sterile water or normal saline* |  |  |  |
| Describe set-up:   - Machine positioning - Patient positioning - Describe which hand holds probe and which holds needle |  |  |  |
| Obtain sonographic image & freeze: |  |  |  |
| Identify critical anatomy:   - *Latissimus dorsi muscle* - *Serratus anterior muscle* - *Intercostal muscles* - *Ribs* - *Pleura/pleural interface* |  |  |  |
| Describe additional relevant anatomy (not necessarily visualized): | - | - | - |
| Describe technique:   - Needle approach - Desired needle tip location - Anticipated anesthesia hydrodissection |  |  |  |
| Describe risks:   - *Pneumothorax* |  |  |  |

| **Fascia Iliaca Block – Traditional View** | **Independently** | **Prompted** | **Unable** |
| --- | --- | --- | --- |
| Identify common indications^10^:   - *Analgesia for hip fracture* - *Femur fracture* |  |  |  |
| Describe anticipated area of anesthesia:   - *Lateral thigh* - *Anterior thigh* - *Medial thigh* - *Medial knee* - *Medial leg* - *Medial ankle* |  |  |  |
| Describe anesthetic used:   - *Generally long-acting anesthesia* - *~20cc of anesthesia combined with 20cc of sterile water or normal saline* |  |  |  |
| Describe set-up:   - Machine positioning - Patient positioning - Describe which hand holds probe and which holds needle |  |  |  |
| Obtain sonographic image & freeze: |  |  |  |
| Identify critical anatomy:   - *Femoral nerve* - *Femoral artery* - *Femoral vein* - *Fascia lata* - *Fascia iliaca* - *Iliopsoas muscle* - *Sartorius muscle* |  |  |  |
| Describe additional relevant anatomy (not necessarily visualized):   - *Lateral femoral cutaneous nerve* - *Obturator nerve* |  |  |  |
| Describe technique:   - Needle approach - Desired needle tip location - Anticipated anesthesia hydrodissection |  |  |  |
| Describe risks:   - *Vascular injection* - *LAST* |  |  |  |

| **Fascia Iliaca Block – Bowtie View** | **Independently** | **Prompted** | **Unable** |
| --- | --- | --- | --- |
| Identify common indications^10^:   - *Analgesia for hip fracture* - *Femur fracture* |  |  |  |
| Describe anticipated area of anesthesia:   - *Lateral thigh* - *Anterior thigh* - *Medial thigh* - *Medial knee* - *Medial leg* - *Medial ankle* |  |  |  |
| Describe anesthetic used:   - *Generally long-acting anesthesia* - *~20cc of anesthesia combined with 20cc of sterile water or normal saline* |  |  |  |
| Describe set-up:   - Machine positioning - Patient positioning - Describe which hand holds probe and which holds needle |  |  |  |
| Obtain sonographic image & freeze: |  |  |  |
| Identify critical anatomy:   - *ASIS* - *Internal oblique muscle* - *Iliopsoas muscle* - *Sartorius muscle* - *Fascia iliaca* |  |  |  |
| Describe additional relevant anatomy (not necessarily visualized): |  |  |  |
| Describe technique:   - Needle approach - Desired needle tip location - Anticipated anesthesia hydrodissection |  |  |  |
| Describe risks:   - *Vascular injection* - *LAST* |  |  |  |

| **Femoral Nerve Block** | **Independently** | **Prompted** | **Unable** |
| --- | --- | --- | --- |
| Identify common indications^8^:   - *Analgesia for hip fracture* - *Femur fracture* |  |  |  |
| Describe anticipated area of anesthesia^7, 8^:   - *Anterior thigh* - *Medial knee* - *Medial leg* - *Medial ankle* |  |  |  |
| Describe anesthetic used:   - *Depends on why you’re performing the block; generally short-acting* - *~5-10cc* |  |  |  |
| Describe set-up:   - Machine positioning - Patient positioning - Describe which hand holds probe and which holds needle |  |  |  |
| Obtain sonographic image & freeze: |  |  |  |
| Identify critical anatomy:   - *Femoral nerve* - *Femoral artery* - *Femoral vein* - *Fascia lata* - *Fascia iliaca* - *Iliopsoas muscle* - *Sartorius muscle^10^* |  |  |  |
| Describe additional relevant anatomy (not necessarily visualized): |  |  |  |
| Describe technique:   - Needle approach - Desired needle tip location - Anticipated anesthesia hydrodissection |  |  |  |
| Describe risks: |  |  |  |

| **Saphenous Nerve Block** | **Independently** | **Prompted** | **Unable** |
| --- | --- | --- | --- |
| Identify common indications:   - None specific! | - | - | - |
| Describe anticipated area of anesthesia^8^:   - *Medial knee* - *Medial leg* - *Medial ankle* |  |  |  |
| Describe anesthetic used:   - *Depends on why you’re performing the block; generally short-acting* - *~5-10cc* |  |  |  |
| Describe set-up:   - Machine positioning - Patient positioning - Describe which hand holds probe and which holds needle |  |  |  |
| Obtain sonographic image & freeze: |  |  |  |
| Identify critical anatomy^11^:   - *Femoral artery* - *Femoral vein* - *Vastus medialis muscle* - *Adductor longus muscle* - *Sartorius muscle* |  |  |  |
| Describe additional relevant anatomy (not necessarily visualized):   - *Saphenous nerve* |  |  |  |
| Describe technique:   - Needle approach - Desired needle tip location - Anticipated anesthesia hydrodissection |  |  |  |
| Describe risks: |  |  |  |

| **Sciatic Nerve Block** | **Independently** | **Prompted** | **Unable** |
| --- | --- | --- | --- |
| Identify common indications:   - No specific indications! | - | - | - |
| Describe anticipated area of anesthesia^12^:   - *Leg except medial* - *Dorsal surface of foot* - *Plantar surface of foot* - *(NOT medial leg and NOT superior to the knee anteriorly)* |  |  |  |
| Describe anesthetic used:   - *Depends on why you’re performing the block; generally short-acting* - *~10cc* |  |  |  |
| Describe set-up:   - Machine positioning - Patient positioning - Describe which hand holds probe and which holds needle |  |  |  |
| Obtain sonographic image & freeze: |  |  |  |
| Identify critical anatomy:   - *Sciatic nerve* - *Tibial nerve* - *Common peroneal nerve (aka common fibular nerve)* |  |  |  |
| Describe additional relevant anatomy (not necessarily visualized):   - *Popliteal artery* - *Popliteal vein* |  |  |  |
| Describe technique:   - Needle approach - Desired needle tip location - Anticipated anesthesia hydrodissection |  |  |  |
| Describe risks: |  |  |  |

| **Tibial Nerve Block** | **Independently** | **Prompted** | **Unable** |
| --- | --- | --- | --- |
| Identify common indications:   - *Sole of foot lacerations* | - | - | - |
| Describe anticipated area of anesthesia:   - *Plantar aspect of the foot* |  |  |  |
| Describe anesthetic used:   - *Depends on why you’re performing the block; generally short-acting* - *~5cc* |  |  |  |
| Describe set-up:   - Machine positioning - Patient positioning - Describe which hand holds probe and which holds needle |  |  |  |
| Obtain sonographic image & freeze: |  |  |  |
| Identify critical anatomy^16^:   - *Medial malleolus* - *Tibial nerve* - *Posterior tibial artery* - *Posterior tibial veins* |  |  |  |
| Describe additional relevant anatomy (not necessarily visualized): |  |  |  |
| Describe technique:   - Needle approach - Desired needle tip location - Anticipated anesthesia hydrodissection |  |  |  |
| Describe risks:   - *Arterial injection* |  |  |  |

**References:**

1. <https://www.acepnow.com/article/pain-control-using-ultrasound-guided-superficial-cervical-plexus-block/>
2. <https://www.nysora.com/techniques/upper-extremity/intescalene/ultrasound-guided-interscalene-brachial-plexus-block/>

1. <https://www.nysora.com/regional-anesthesia-for-specific-surgical-procedures/upper-extremity-regional-anesthesia-for-specific-surgical-procedures/anesthesia-and-analgesia-for-elbow-and-forearm-procedures/ultrasound-guided-supraclavicular-brachial-plexus-block/>
2. <https://www.acepnow.com/article/perform-ultrasound-guided-forearm-nerve-blocks-provide-non-drug-pain-relief-acute-injuries/>

1. <https://www.nysora.com/regional-anesthesia-for-specific-surgical-procedures/thorax/pectoralis-serratus-plane-blocks/>

1. <https://www.acepnow.com/article/ultrasound-guided-serratus-anterior-plane-block-can-help-avoid-opioid-use-patients-rib-fractures/>
2. <https://www.nysora.com/techniques/lower-extremity/femoral/femoral-nerve-block/>

1. <https://www.nysora.com/techniques/lower-extremity/ultrasound-guided-femoral-nerve-block/>
2. <https://www.acep.org/sonoguide/femoral_nerve_block.html>

1. <https://www.nysora.com/regional-anesthesia-for-specific-surgical-procedures/lower-extremity-regional-anesthesia-for-specific-surgical-procedures/ultrasound-guided-fascia-iliaca-block/>
2. <https://www.nysora.com/regional-anesthesia-for-specific-surgical-procedures/lower-extremity-regional-anesthesia-for-specific-surgical-procedures/foot-and-anckle/ultrasound-guided-saphenous-subsartorius-adductor-canal-nerve-block/>
3. <https://www.nysora.com/regional-anesthesia-for-specific-surgical-procedures/lower-extremity-regional-anesthesia-for-specific-surgical-procedures/foot-and-anckle/ultrasound-guided-sciatic-nerve-block-2/>
4. <https://www.nysora.com/techniques/lower-extremity/sciatic-nerve-block/>
5. <https://www.acep.org/sonoguide/posterior_tibial_nerve_block.html>
6. <https://www.nysora.com/regional-anesthesia-for-specific-surgical-procedures/lower-extremity-regional-anesthesia-for-specific-surgical-procedures/foot-and-anckle/ankle-block/>
7. <http://highlandultrasound.com/posterior-tibial-block>
